# Homophilic binding of the neural cell adhesion molecule CHL1 regulates development of ventral midbrain dopaminergic pathways

**DOI:** 10.1038/s41598-017-09599-y

**Published:** 2017-08-24

**Authors:** W. F. Alsanie, V. Penna, M. Schachner, L. H. Thompson, C. L. Parish

**Affiliations:** 1 The Florey Institute of Neuroscience and Mental Health, The University of Melbourne, Melbourne, Australia; 2 The Department of Medical Laboratories, The Faculty of Applied Medical Sciences, Present Address: Taif University, Taif, Saudi Arabia; 3 Keck Center for Collaborative Neuroscience and Department of Cell Biology and Neuroscience, Rutgers University, New Brunswick, NJ USA

## Abstract

Abnormal development of ventral midbrain (VM) dopaminergic (DA) pathways, essential for motor and cognitive function, may underpin a number of neurological disorders and thereby highlight the importance of understanding the birth and connectivity of the associated neurons. While a number of regulators of VM DA neurogenesis are known, processes involved in later developmental events, including terminal differentiation and axon morphogenesis, are less well understood. Recent transcriptional analysis studies of the developing VM identified genes expressed during these stages, including the cell adhesion molecule with homology to L1 (*Chl1*). Here, we map the temporal and spatial expression of CHL1 and assess functional roles of substrate-bound and soluble-forms of the protein during VM DA development. Results showed early CHL1 in the VM, corresponding with roles in DA progenitor migration and differentiation. Subsequently, we demonstrated roles for CHL1 in both axonal extension and repulsion, selectively of DA neurons, suggestive of a role in guidance towards forebrain targets and away from hindbrain nuclei. In part, CHL1 mediates these roles through homophilic CHL1-CHL1 interactions. Collectively, these findings enhance our knowledge of VM DA pathways development, and may provide new insights into understanding DA developmental conditions such as autism spectrum disorders.

## Introduction

Ventral midbrain (VM) dopaminergic (DA) neurons, forming the nigrostriatal and mesocorticolimbic pathways, play critical roles in motor and cognitive function. Dysfunction of these neurons is associated with a number of neurological conditions, including schizophrenia and autism, that may be underpinned by abnormal developmental (for review see ref. 1), as well as progressive neurodegenerative disorders such as Parkinson’s disease (PD). Consequently, an enhanced knowledge of the processes involved in the birth and connectivity of these neurons may shed new light on understanding disease pathogenesis.

The development of midbrain dopamine neurons commences with proliferating progenitors within the VM migrating radially from the ventricular surface into the intermediate zone (IZ), where they become post-mitotic NURR1+ neuroblasts. These neuroblasts continue radial migration into the marginal zone (MZ) were one subset terminally differentiate into tyrosine hydroxylase (TH+) expressing DA neurons of the ventral tegmental area (VTA), while another subset undergo subsequent tangential migration towards the basal plate to become DA neurons of the substantia nigra (SN)^2–4^. As such, the first TH+DA neurons are reported to appear in the MZ of the developing mouse VM at embryonic day (E)10.5, with DA neurogenesis ceasing around E14.5^5, 6^. Following their birth, polarized VM DA neurons extend neurites dorsally within the midbrain, prior to deflecting their axons rostrally along the medial forebrain bundle^7, 8^. These axons then enter the ventromedial, and subsequently dorsolateral ganglionic eminence (developing striatum), as well as the overlying cortex, to form the nigrostriatal and mesocorticolimbic pathways, respectively (for review see ref. 9). Finally, axonal remodeling and synaptogenesis continues into early postnatal life^7, 8^.

Although several regulators of these developmental processes have been elucidated, including the early DA fate determinant genes (*Ngn2*, *Lmx1a/b* and *Mash1)*, maturation genes (*Wnt5a*, *TH* and *Pitx3*) as well as axonal plasticity genes (*Netrin1* and *Wnt5a/7a*), these known genes fail to account for all processes specific to VM DA development, see reviews-^2, 8, 10^. Recent transcriptional analysis studies by us and others, involving the temporal and/or spatial isolation of DA progenitors, have revealed a number of additional genes that may be important in VM DA development^11, 12^. One such gene, common to both studies, was the neural cell adhesion protein Close homolog to L1 (*Chl1*) - a gene already identified for developmental roles in the fore- and hindbrain^13–17^. Here we describe the temporal and spatial expression of CHL1 within the developing VM and, through iterative *in vitro* studies and assessment of CHL1 deficient mice, link patterns of expression to key events in establishment of the VM DA pathways including DA differentiation, neuroblast migration and axon morphogenesis.

## Methods and Materials

### Animals

This study followed the guidelines of the Australian National Health and Medical Research Council’s Published Code of Practice for the Use of Animals in Research, and the experiments were approved by the Florey Institute of Neuroscience and Mental Health animal ethics committee (#14–042).

Embryos were isolated from time-mated Swiss mice (Animal Resource Centre, Australia). Animals were time mated overnight and visualisation of a vaginal plug on the following morning was considered as embryonic day (E) 0.5. CHL1 deficient (CHL1−/−) embryos and their littermate wildtype controls, maintained on a C57BL/6 background, were obtained from Professor M. Schachner (University of Hamburg, Germany)^15^.

### Quantitative real-time PCR

Quantitative PCR (qPCR) was used to examine the temporal expression of *Chl1* within the developing VM. In brief, the VM was isolated from E10.5, E12.5, E14.5 and E18.5 embryos (4 litters per age) as previously described^7, 18^. The VM was additionally isolated from tyrosine hydroxylase green fluorescent protein (TH-GFP+) embryos at E12.5^19^, to establish whether *Chl1* was expressed on DA neurons (FACS isolated THGFP+ cells) or non-dopaminergic neurons (THGFP− cells) within the VM (n = 4 embryos per FACS preparation, repeated on 4 litters). For all samples RNA was isolated from using RNeasy Micro kit (Qiagen) and reverse transcribed using a SuperScript® VILO™ cDNA Synthesis Kit. qPCR performed using SYPR GreenER qPCR SuperMix Universal (Invitrogen) on a Rotor-Gene 6000 thermocycler (QIAGEN) and analyzed using the comparative ΔΔCT method^20^. Oligonucleotide sequences were as follows:

*Hprt* forward, 5′-CTTTGCTGACCTGCTGGATT-3′

*Hprt* reverse, 5′-TATGTCCCCCGTTGACTGAT-3′

*Chl1* forward, 5′-TGGAATTGCCATTATGTGGA-3′

*Chl1* reverse, 5′-CACCTGCACGTATGACTGCT-3′

### Immunohistochemistry

Embryos were fixed in 4% paraformaldehyde (2–8 hrs, dependent on developmental age), cryopreserved in 20% sucrose and coronally sectioned (16 μm, 1:10 series). For primary cultures and explant assays, cells were fixed with 4% paraformaldehyde, rinsed and stored in PBS with 0.03% sodium azide.

Immunohistochemistry was performed using previously described methods^21^. Antibodies used were as follows: goat anti-CHL1 (1:500, R&D Systems), rabbit anti-NURR1 (1:200, Santa Cruz), rabbit anti-TH (1:800, PelFreez), chicken anti-TH (1:400, Abcam), mouse anti-TUJ1 (1:15000, Promega), and chicken anti-TUJ1 (1:200, Millipore). Appropriate secondary antibodies, donkey 488, 555 and 647 Alexaflour (Jackson ImmunoResearch Laboratories), were used at a dilution of 1:400. Images were captured using fluorescence microscope (Zeiss Axio Observer Z1 or Zeiss 780 confocal microscrope).

### Cell migration and axon chemotaxis assays

E10.5 VM neurospheres and E11.5 VM explants were generated using previously described methods^7, 21^. Heparin acrylic beads (Sigma), pre-incubated for 24 hrs in either PBS (control) or CHL1 (10μg/ml, R&D Systems), were attached to poly-D-lysine coated coverslips using rat tail collagen (2.1 mg/ml, Roche). The VM tissue (neurosphere or explant) was positioned adjacent to the beads, at a distance of approximately 300μm. Subsequently, both the VM tissue and beads were encapsulated in collagen gel and cultured in the presence of N2 media (consisting of a 1:1 mixture of F12 and MEM supplemented with 15 mM HEPES buffer, 1 mM glutamine, 6 mg/ml glucose, 1 mg/ml bovine serum albumin and N2 supplement) for 72 h, prior to fixation (4% paraformaldehyde, 20 min) and immunostaining.

### Ventral midbrain primary culture

VM primary cultures were performed on E11.5, E12.5 and E14.5 litters, as previously described^7^. CHL1 recombinant protein (1μg/ml) was added directly to the cultures at the time of cell plating, or tethered to culture plates prior to cell seeding (to assess the function of soluble and membrane-bound CHL1, respectively). For CHL1 protein attachment, poly-D-lysine coated plates were incubated overnight with a His-tag antibody (FC). The following day, the FC antibody was removed, plates blocked with 1% BSA (30 min, 37 °C) and subsequently CHL1 recombinant protein (2 μg/ml) or 1% BSA (control) added to the wells. Plates were incubated overnight (37 °C) and rinsed the following morning prior to seeding of VM primary neurons. Primary neurons and explants were cultured for 3 days *in vitro* (DIV), prior to quantitative assessments.

### Quantification

Differentiation was assessed in E11.5 cultures immunolabeled with TH, TUJ1, and counter-stained with 4′,6-diamidino-2-phenylindole (DAPI). Counts were made of total DAPI, TUJ and TH labeled cells from 10 fields of view (20× magnification), from 3 technical replicates and 4 independent cultures.

Migration of dopaminergic progenitors was assessed in 30 neurospheres per culture (repeated in 4 independent cultures). Images of each neurosphere were captured and the sphere split in half, parallel to the location of the beads (see Fig. 2A), with total TH+ cells migrating away from the proximal and distal sides of the sphere quantified and expressed as a relative proximal:distal ratio, with 1 reflective of even migration of cells from both sides of neurosphere.

Neurite morphology was examined on E12.5 VM primary cultured neurons. Thirty dopaminergic (TH+/TUJ+) and 30 non-dopaminergic (TH−/TUJ+) neurons were assessed for each condition from each culture (n = 4 cultures). For each neuron, assessments included: total neurites, number of branches, total neurite length as well as dominant neurite length (indicative of the axon^22^).

### Analysis of CHL1 knockout mice

E12.5, E14.5 and E18.5 knockout (﻿﻿KO) embryos as well as their wildtype (﻿WT) littermates were sectioned as described previously. To assess the migration of dopaminergic neurons *in vivo*, E12.5 and E14.5 sections, containing TH+ immunolabeled neurons and counterstained with 4′,6-diamidino-2-phenylindole (﻿DAPI),﻿ were imaged and the VM divided into intermediate and marginal zones (IZ and MZ respectively), using DAPI cell density to delineate the boarder of the 2 regions. The marginal zone was subsequently divided into 3 equal portions from the midline (each 250μm wide) and termed MZ1 (most medial), MZ2, and MZ3 (most lateral), see Fig. 2G for delineations. Total TH+ cells within the VM as well as within each of the defined zones (IZ, MZ1, MZ2 and MZ3) were quantified to identify radial (from IZ to MZ) and tangential (MZ1-MZ3) migration defects. Assessment of midbrain dopaminergic pathway defects were made from E12.5 (sagittal), E14.5 (coronal) and E18.5 (sagittal) sections obtained from CHL1 KO embryos and their littermate controls, using previously described methods^7^. In brief, the presence of gross defects in TH+ axonal projections of E12.5 and E18.5 CHL1 KO embryos (including orientation of axonal growth, targeting and/or elongation) was assessed (n = 3/age/genotype), as well as total TH+ neurites within the medial forebrain bundle and degree of axonal fasciculation, (n = 4/genotype).

### Assessment of mouse embryonic stem cell-derived DA neurons

The mouse embryonic stem cell (ESC) line, E14TG2a (ATCC, USA), was maintained undifferentiated on gelatin-coated culture plates in serum-free 2i medium (consisting of DMEM/F12, 1× N2 supplement, 1× B27 –vitamin A, 1× penicillin/streptomycin (P/S), 1× glutamax, 1× non-essential amino acids, 0.11 mM beta-mercaptoethanol, 2000 IU/ml LIF, 1 mM MEK inhibitor PD0325901 (Stemgent) and 3 mM GSK3 inhibitor CHIR9902 (Stemgent)). All reagents were purchased from GIBCO unless stated otherwise.

To initiate VM differentiation, ESCs, seeded at a density of 7000 cells/cm^2^, were cultured in gradient serum replacement medium (SRM) and N2 medium, supplemented with 200 nM LDN193189 (Tocris), (day 0: 100% SRM; day 1: 75% SRM:25% N2; day 2: 50% SRM:50% N2; day 3: 25%SRM:75% N2; day 4–6: 100% N2 media). SRM consisted of Knockout DMEM, 1× P/S, 1× glutamax, 1× NEAA, 0.11 mM beta-mercaptoethanol and 15% knockout serum. N2 medium components included DMEM/F12, 1× P/S, 1× glutamax, 1× NEAA, 0.11 mM beta-mercaptoethanol, 1× insulin-transferrin-selenium-sodium pyruvate supplement (ITS-A) and 1× N2 supplement. To promote ventralization of cells, cultures were supplemented with sonic hedgehog (SHH, 200 ng/ml, R&D) and puromorphomine (PM, 2μM, Stemgent), and caudalized by the addition of fibroblast factor 8 (FGF8, 25 ng/ml, R&D) from days 1–7. Media was further supplemented with Wnt activator CHIR9902 (0.3μM, Stemgent) from day 2–7, as well as FGF2 (20 ng/ml, Peprotech) on day 3–7. On day 7, the differentiating cells were split (1:1) using accutase and replated on either: (i) PDL-coated plates with media supplemented with CHL1 (to examine the effects of soluble CHL1) or, (ii) FC or CHL1-coated plates (as described earlier, to determine the effects of bound CHL1). At the time of replating, cells were switched to a maturation media (consisting of 1:1 mixture of DMEM/F12 and Neurobasal medium, 1× P/S, 1× glutamax, 1× NEAA, 0.11 mM beta-mercaptoethanol, 1× ITS-A, 1× N2 supplement and 1× B27+ vitamin A supplement) supplemented with GDNF (30 ng/ml, R&D), BDNF (30 ng/ml, R&D), ascorbic acid (0.2 mM, Sigma-Aldrich) and 10 μM DAPT (Tocris).

### Statistical analysis

Statistical analysis was done using GraphPad Prism 6. Student t-test or one-way ANOVA with Tukey post-hoc tests were used to identify statistically significant changes. All quantitative data are expressed as means ± SEM with significance set at p < 0.05.

## Results and Discussion

Understanding the intricate and complex sequence of events that lead to the birth and connectivity of neurons during development has been, and remains, an ongoing quest. This is particularly true for the DA pathways that originate in the ventral midbrain and innervate distant cortical and striatal targets. Alterations in the connectivity of these pathways, and associated dopamine neurotransmission, underlies a number of neurological disorders, including Parkinson’s disease, addiction and schizophrenia^1^. The identification of novel genes involved in the development of these dopamine pathways may aide in our understanding of these disorders and/or establishment of new therapies. Whilst a number of genes and proteins have been identified, here we characterize the diverse roles of the cell adhesion molecule, close homolog to L1, CHL1, in the development of VM DA neurons.

Elsewhere in the CNS, the expression and function of CHL1 has been well characterized. Within the developing olfactory bulbs, cortex, hippocampus and cerebellum, CHL1 has been shown to be expressed on both neurons and surrounding glia (including astrocytes and oligodendrocytes)^13–17^. The temporo-spatial patterns of expression, combined with numerous loss and gain-of-function studies, have identified diverse roles for CHL1 in development of forebrain and hindbrain regions including positively influencing cell migration, orientation, neurite elongation and synaptogenesis, while negatively regulating proliferation and differentiation^13–17^. By contrast, the expression and function of CHL1 in the VM remains poorly characterized.

In support of possible roles in CHL1 in DA development and homeostasis in mature neuronal function, CHL1 expression has already been confirmed within the developing VM^12^, and more specifically localized on DA progenitors^11^. Furthermore, *Chl1* has been linked to several neurological conditions, including schizophrenia^23–25^, depression^26^, and autism^27^; with these disorders already characterized by altered DA transmission.

### CHL1 displays a temporo-spatial expression pattern suggestive of a role in development of VM DA neurons

**Figure 1:**
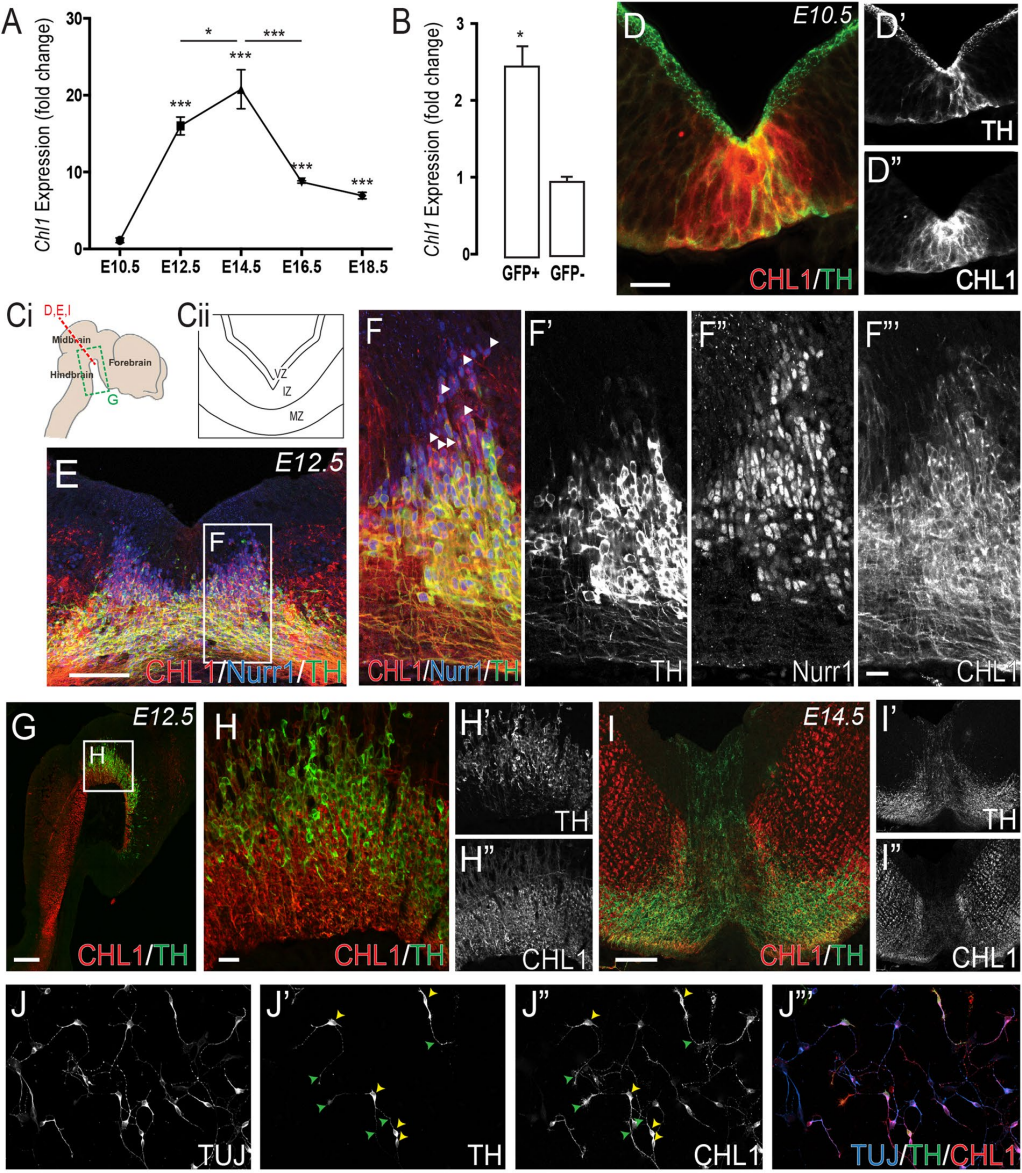
Temporal and spatial expression of CHL1 within the VM during periods of DA neurogenesis and axon morphogenesis. (**A**) Quantitative PCR showing temporal expression of CHL1 within the developing VM, and (**B**) selective expression within DA (THGFP+) and non-DA (THGFP−) cells, isolated by flow cytometry from the VM of THGFP E12.5 embryos. (**Ci**) Sagittal and (**Cii**) coronal schematic of the developing brain illustrating region of interest depicted in panels D–I. (**D**) Coronal section of the developing VM illustrating overlapping TH and CHL1 expression at E10.5. (**E**,**F**) At E12.5, CHL1 was expressed in intermediate and marginal zone of the VM, overlapping with NURR1+ and TH+ cells. (**G**,**H**) E12.5 sagittal images illustrating strong CHL1 expression within, and caudal to the VM, yet absent from the forebrain. (**I**) By E14.5 CHL1 was broadly expressed within the intermediate and marginal zone of the VM basal plate, and weakly expressed within the floorplate. (**J**) E14.5 VM primary culture illustrating the expression of TUJ, (**J’**) TH, (**J”**) CHL1 and (**J’”**) Merged. Note the expression of CHL1 on both TH+ DA soma (yellow arrowsheads) and their axons (green arrowhead). Data represents mean ± SEM, n = 3–5. *p < 0.05, ***p < 0.001. Scale bars: E,G,I = 300 μm D,F,H,I = 50 μm.

Here, a detailed analysis of the temporal and spatial expression of CHL1 within the developing VM was performed to establish its presence and possible role/s in DA neurogenesis and axon morphogenesis. qPCR revealed a significant upregulation in *Chl1* expression within the VM at E10.5 to E12.5 (compared to E10.5 whole embryo), corresponding with periods of DA progenitor migration, differentiation and initiation of neurite growth (Fig. 1A). By E14.5, a period when DA axons approach their forebrain targets, *Chl1* expression peaked, prior to being significantly downregulated at E16.5. Fluorescence activated cell sorting for GFP+ and GFP− cells, isolated from the VM of E12.5 TH−GFP embryos, confirmed *Chl1* expression on both VM DA neurons (TH-GFP+), as previously reported^11^, as well as other surrounding cell populations within the VM (TH-GFP− cells), Fig. 1B.

Complementary immunohistochemical analysis enabled a more detailed assessment of the spatial expression of CHL1 within the developing VM. At E10.5 CHL1 was strongly expressed at the ventral midline, through the thickness of the neuroepithelium, and overlapped with early born TH+ VM DA neurons (Fig. 1D). By E12.5, CHL1 expression was not restricted to the ventral midline, but rather more broadly expressed into the adjacent basal plate, with CHL1 absent from the ventricular zone (VZ), yet present in both the Nurr1+ intermediate zone (IZ) and Nurr1+/TH+ marginal zone (MZ), Fig. 1E,F, Refer to Figure 1Cii for schematic illustration of VM zones. Sagittal sections highlighted CHL1 expression throughout the rostro-caudal axis of the VM, overlapping with TH neurons, with expression extending caudally into the hindbrain and spinal cord (Fig. 1G). By E14.5, CHL1 was weakly expressed within the medial floorplate, with higher expression in the lateral basal plate (Fig. 1H). Primary cultures of E14.5 VM cells highlighted maintained expression of CHL1 on the soma (yellow arrows, Fig. 1J’,J”) and axons (green arrows, Fig. 1J’,J”) of TH+ neurons. These temporal and spatial expression patterns within the embryonic VM suggest possible roles in DA development.

### CHL1 is important for the migration of VM DA neuroblasts

**Figure 2:**
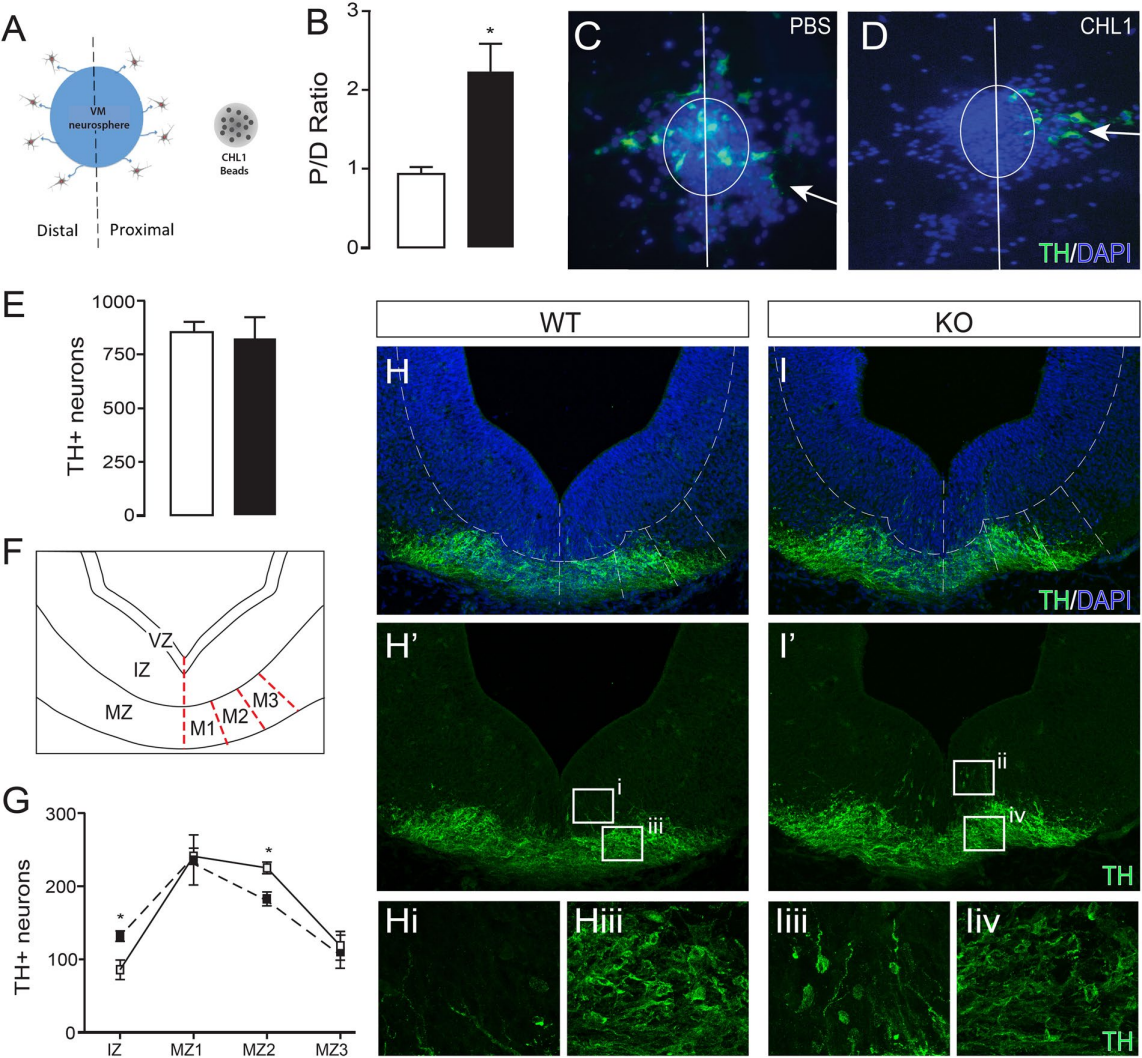
CHL1 contributes to the radial and tangential migration of VM DA neurons. (**A**) Schematic of migration assay illustrating a VM neurosphere adjacent to PBS or CHL1-loaded heparin acrylic beads. Within this assay the number of TH+DA neurons migrating towards (proximal) and away (distal) from the beads were assessed. (**B**) DA neurons were attracted to CHL1 (black bar), with significantly more TH+ cells observed emanating from the proximal side of the VM neurosphere, (i.e. proximal:distal (P/D) ratio > 1), compared to culturing in the presence of PBS-loaded beads (White bar). Representative VM neurospheres cultured in the presence of (**C**) PBS- and (**D**) CHL1-loaded beads. Arrow indicates the direction of ligand signal (i.e. bead) relative to sphere. (**E**) Quantification of TH+ neurons in the VM of E12.5 CHL1 WT (white) and CHL1 KO (black) embryos. (**F**) Schematic of the VM illustrating the radial zones (ventricular, VZ; intermediate, IZ; and marginal, MZ) and subdivision of the marginal zones (M1, M2, M3). (**G**) Quantification of TH+ neurons within the IZ and M1-M3 areas of the marginal zone. Note the increase in TH+ cells in the IZ of CHL1 KO embyros (black symbols) compared to WT (white symbols), reflective of a radial migration defect, as well as the decrease in TH+ cells in the M2 area of the MZ, suggestive of changes in tangential migration. Representative images showing the distribution of TH+ neurons in the VM of CHL1 WT (**H**), and KO (I) embryos at E12.5. Data represents mean ± SEM, *p < 0.05.

Within the developing VM, neuroblasts undergo initial radial migration, from the VZ to the MZ, and subsequently migrate tangentially from the midline to form two principal nuclei - the medial ventral tegmental area and the more laterally placed substantia nigra pars compacta^6^. While recent studies have demonstrated CXCR4/CXCL12 and Reelin signaling contributing to these radial and tangential migratory events, respectively^28–30^, spatial expression of CHL1, on NURR1+ DA progenitors and in fibres extending ventrally from the IZ into the MZ (Fig. 1), as well as laterally within the MZ, suggested this protein may also play a role in neuroblasts migration. CHL1, released from heparin acrylic beads positioned adjacent to VM-derived neurospheres (Fig. 2A), provided chemoattractive cues to DA neuroblasts, with a significant increase in the number of TH+ neurons observed migrating from the proximal half of the neurosphere (Proximal to Distal ratio, (P/D): PBS, 0.96 ± 0.11 and CHL1, 2.21 ± 0.36, Fig. 2B–D). Confirming the role of CHL1 in VM DA migration, and to more specifically determine its role in radial and/or tangential migration, the distribution of TH-positive neurons within the VM was assessed in CHL1 WT and KO littermates. While total number of TH+ neurons in the VM was not different between KO and WT embryos (Fig. 2E), significantly more cells were present in the IZ of KO embryos, indicative of a radial migration defect (Fig. 2F,H,I). Furthermore, significantly more TH+ cells were observed within the MZ2 region of the VM, suggestive of a disruption in tangential migration. These findings support previous studies demonstrating the role of CHL1-mediated cell migration in the neocortex and cerebellum^13, 17^. Examination of the distribution of TH+ neurons later in development (E14.5, and corresponding to the end of DA neurogenesis) revealed persistent migratory defects, with a residual population of TH+ neurons present within the IZ of CHL1 KO mice, that were not observed in WT littermates, Supplementary Figure 1. It remains to be determined whether the migration defects observed in the CHL1 deficient mice were the consequence of: (i) soluble CHL1 acting homophilically with CHL1 on DA neurons, (ii) soluble CHL1 acting heterophilically via other L1 family adhesion proteins or integrins on DA neurons, noting previously observed DA migration defects in L1 deficient mice^31^, or (iii) whether bound CHL1, present on radiating and tangential (DA or non-DA) fibers within the VM, also contribute to the final positioning of DA neurons during development.

### CHL1 promotes the differentiation of VM DA neurons through homophilic interactions

**Figure 3:**
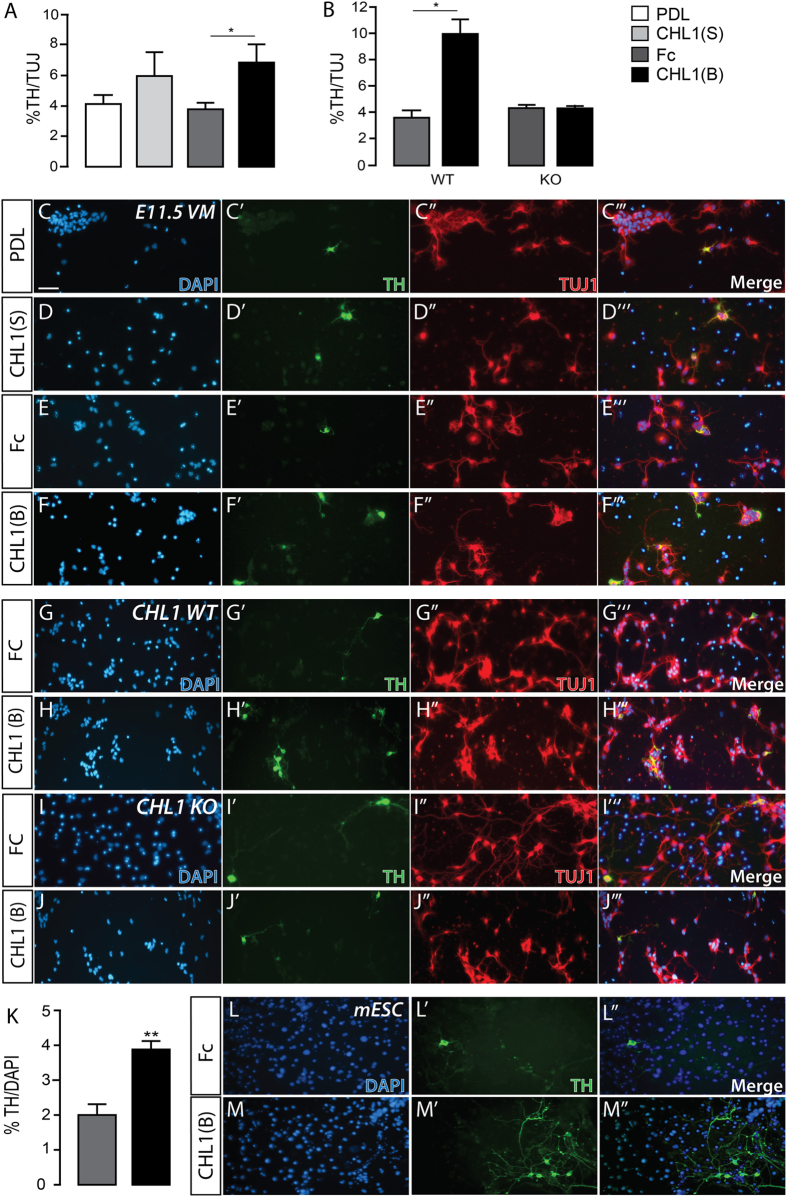
Homophilic CHL1-CHL1 binding promotes DA differentiation of VM neurons. (**A**) Proportion of TH+/TUJ+ neurons in E11.5 VM cultures from Swiss mice grown on PDL (white), and treated with soluble (CHL1(S), light grey), or cultured on tethered Fc (Fc, dark grey), together with surface-bound CHL1 (CHL1(B), black). (**B**) %TH+/TUJ+ neurons in CHL1 WT and CHL1 KO cultures with and without CHL1(B). Note the failure of CHL1(B) to enhance DA differentiation in KO mice, suggestive that a homophilic CHL1-CHL1 interaction regulated these changes in WT mice. (**C**–**F**) Representative photomicrographs of E11.5 VM cultures from Swiss mice in the presence and absence of CHL1(S) and CHL1(B), including appropriate PDL and Fc controls, and stained for DAPI, TH, TUJ (and merged images). Images of VM cultures from CHL1 WT (**G**,**H**), and CHL1 KO mice (**I**,**J**) in the presence and absence of soluble and bound CHL1. (**K**) CHL1(B) similarly enhanced the proportion of TH+ neurons within mouse ESC cultures, differentiated towards a VM fate. (**L**,**M**) Example images of differentiated mESC cultured on Control (Fc) and CHL1(B) plates, illustrating the increase TH+ neurons.Data represents mean ± SEM, n = 3–4. *p < 0.05, **p < 0.01.

The temporal expression of CHL1 coincided with the period of DA neurogenesis (spanning from E10.5–14.5, Fig. 1A), with *Chl1* expressed on both DA neurons as well as surrounding cells within the developing VM (Fig. 1B). This suggested that soluble and/or bound forms of the protein may influence the differentiation of VM progenitors. To examine the effect of CHL1 in this context we treated E11.5 VM primary cultures with soluble CHL1 (CHL1(S), added directly to the media) or bound CHL1 (CHL1(B), tethered to culture plates) and quantified the proportion of TH+ neurons in culture. Note, neither CHL1(S) or CHL1(B) altered total number of DAPI label nuclei or TUJ+neurons (data not shown), suggesting that the protein had no impact on overall survival and/or proliferation of VM neurons. Only substrate-bound CHL1 significantly increased the proportion of TH+ neurons in culture, supportive of a contact mediated mechanism through which CHL1 to DA differentiation (Fig. 3A,C–F). To determine whether CHL1 on neurons directly interacted with bound CHL1 (i.e. homophilic binding) to promote DA maturation, primary cultures were performed from single ventral midbrains, isolated from CHL1 WT and KO embryos (n = 11 and 13, respectively). Again, bound CHL1 significantly increased the proportion of TH+ cells in culture isolated from WT embryos (untreated: 3.8% ± 0.2, CHL1(B): 10.0 ± 1.0), yet failed to increase the proportion of TH+ neurons in CHL1 KO embryo-derived cultures.

Further validating these findings, we demonstrated that bound CHL1 (Fig. 3I–K), but not soluble CHL1 (data not shown), enhanced the yield of TH+ neurons (by 2-fold) in mouse embryonic stem cell cultures differentiated towards a VM fate. Whilst we demonstrate a CHL1-CHL1 homophilic mechanism regulating VM DA differentiation *in vitro*, *in vivo* CHL1 KO mice showed no change in total DA neurons within the VM (Fig. 2E), suggestive of compensation by other adhesion molecules during development. These findings, whilst in contrast to the previously described roles of CHL1 in negatively regulating neuronal differentiation elsewhere in the CNS^14, 16^, suggests CHL1 could be used to increase DA yields in cultures; outcomes that may be of benefits for *in vitro* DA drug screening and cell replacement therapy in PD where large numbers and enrichment for DA neurons is required^32^.

### CHL1 promotes neurites outgrowth and repulsion via homophilic binding

**Figure 4:**
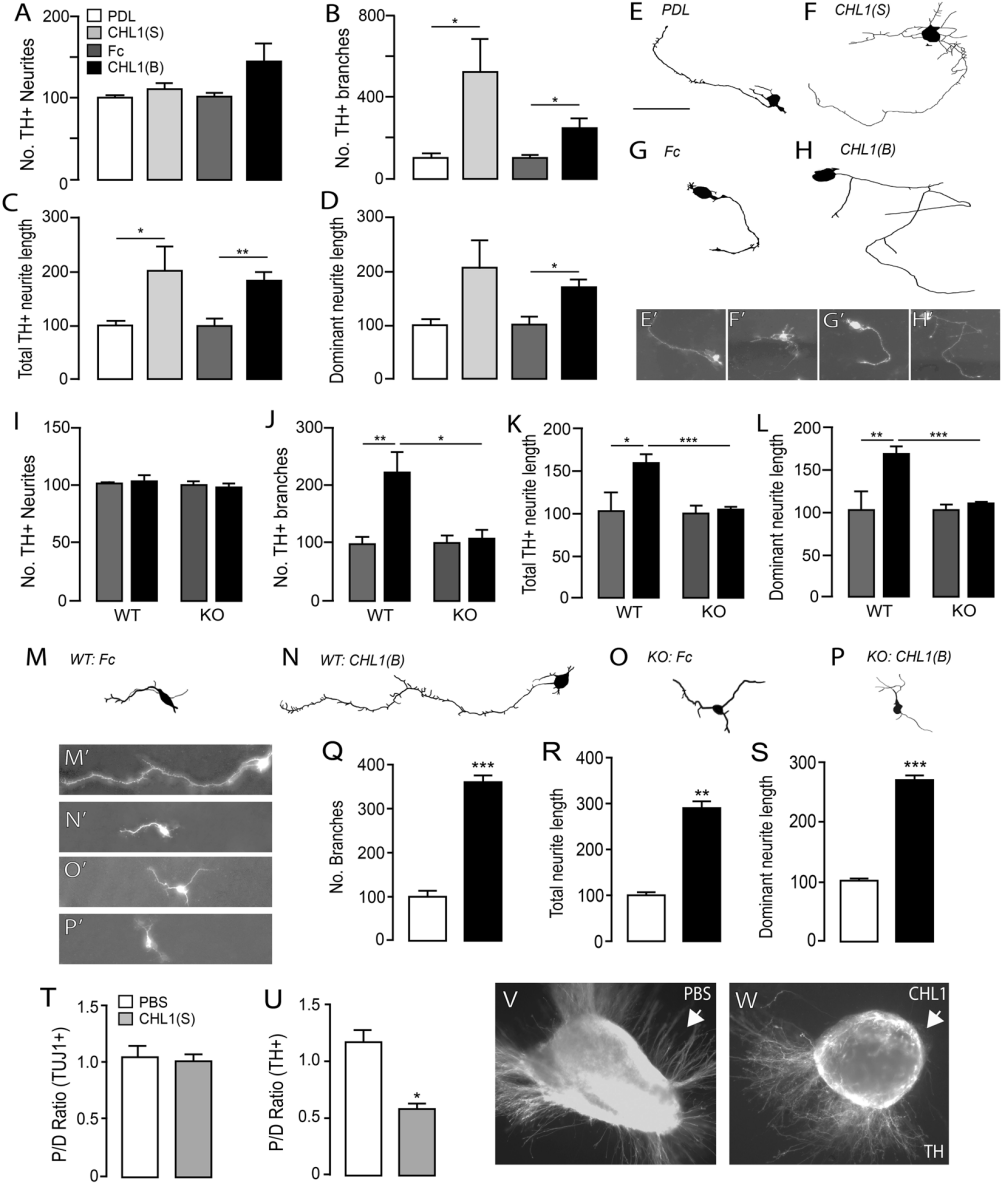
CHL1 influences dopaminergic axon morphogenesis. Soluble and bound CHL1 had no influence DA neurite number (**A**), yet significantly increased neurite branching (**B**), neurite length (**C**), and more specifically CHL1(B) promoted DA axon extension (increased dominant neurite length) (**D**). (**E,H**) Reconstruction of example TH+DA neurons within E12.5 VM cultures in the presence or absence of CHL1 (S or B). (E’-H’) Photomicrographs of neurons reconstructed in (**E**–**H**). (**I**) Quantification of TH+ neurite number, (**J**) TH+ neurite branching, (**K**) total TH+ neurite length and (**L**) TH+ dominant neurite length of E12.5 VM DA neurons from CHL1 KO and littermate cultures, in the presence and absence of CHL1(B). The loss of effect of CHL1(B) on DA neurite morphology suggested CHL1-CHL1 homophilic binding underpinned these responses. Reconstruction of example TH+DA neurons from WT (**M**,**N**), and KO (**O**,**P**) VM cultures in the presence or absence of bound CHL1. (**O’**–**P’**) Corresponding photomicrographs. Similar to primary cultured VM DA neurons, mESC-derived VM DA neurons showed increased (**Q**) number of branches, (**R)** total neurite length and (**S**) dominant neurite length when cultured on substrate-bound CHL1 (black bars), compared to Fc controls (white). (**T**) Assessment of proximal to distal neurite growth from VM explants cultured in the presence of PBS or CHL1-loaded beads illustrating CHL1 had no effect on TUJ+ non-dopaminergic neurites, (**U**) yet significantly repelled TH+ neurites, as revealed by the reduction in P/D ratio. (**V**) Representative photomicrographs of a VM explant illustrating directional growth of TH+ neurites in response to PBS−, and (**W**) CHL1-loaded beads. Note the repulsion of TH+ neurites in response to CHL1. Arrow represent direction of chemotaxic gradient. Data represents mean ± SEM, *p < 0.05.

Previous studies have demonstrated gradient expression of CHL1 influencing neurite growth and orientation within the developing cortex, hippocampus and cerebellum through both homophilic CHL1-CHL1 interactions, as well as heterophilic binding of CHL1 with other integrins and acting as a co-receptor for more classical axon guidance receptors such as Sema3A and EphA7^13, 15, 16, 31, 33–35^. Here we examined the effect of CHL1 on VM DA neurite morphology. In E12.5 VM primary cultures, CHL1 had no effect on TH+ neurite number (Fig. 4A), yet significantly increased neurite branching (CHL1(S): 5-fold; CHL1(B): 2.4-fold) and length (CHL1(S): 2-fold; CHL1(B): 1.8-fold), notably of the dominant neurite (Fig. 4B–H), when compared with appropriate PDL or Fc controls. The reproducibility of these findings (similar fold-changes in neurite length for soluble and bound CHL1, compared to appropriate controls), suggests that these effects were mediated through CHL1-CHL1 homophilic interactions (rather than soluble CHL1 binding with other L1 family proteins or integrins on DA neurons). As such these results were confirmed by our ability to ablate the effect of substrate-bound CHL1 on DA neurite branching and length in primary cultures isolated from CHL1 deficient embryos, compared to wildtype littermates (Fig. 4 I–P).

The influence of CHL1 on TH+ neurons was also demonstrated in mouse ESC-derived VM cultures, where both soluble (data not shown) and substate-bound CHL1 enhanced TH+DA neurite branching, and axonal length (Figs 3J’ and 4Q–S). Furthermore, the effects of soluble and substrate-bound CHL1 on VM primary neurons were specific to TH+DA neurons, with no significant change in neurite branching or increase in neurite length observed in TH−TUJ+ neurons in culture (Supplementary Figure 1). Interestingly, previous studies have suggested that homophilic CHL1 interactions block neurite outgrowth, while CHL1 heterophilic interactions promote the neurites outgrowth^16, 36^. The present findings suggest that homophilic and heterophilic interactions of CHL1 induce different effects based on the origin of the neuronal population.

Finally, we examined the chemotaxic influence of CHL1 on DA neurites (in VM explant cultures isolated from E12.5 embryos and cultured adjacent to PBS− or CHL1-loaded beads). We demonstrated that gradients of CHL1 selectively repelled TH+ neurites (P/D ratio, PBS-beads: 1.17 ± 0.19, CHL1-beads: 0.58 ± 0.08, Fig. 4U–W), whilst bearing no influence on other non-DA neurites (TH−/TUJ+, Fig. 4T). Of relevance, during establishment of the VM DA pathways, DA neurites initially project dorsally prior to being deflected towards their forebrain targets via the medial forebrain bundle. The chemorepulsive effect of CHL1, combined with ventro-dorsal (high expression in the marginal, yet absence from the ventricular zone, Fig. 1F) and caudo-rostral gradients of CHL1 (high expression in the hindbrain but absence in the ventral forebrain), suggests CHL1 may influence the dorsal and subsequent rostral trajectory of TH+axons during establishment of the VM DA pathways. Lack of gross DA pathway defects in CHL1 deficient embryos (at E12.5, E14.5 and E18.5, Supplementary figure 3) suggests other L1-family or integrin proteins are capable of compensating for these developmental events, and/or are supported by other axon guidance cues, such as the Wnt family^7, 37, 38^.

### Concluding remarks

In summary, here we identify a number of novel roles for CHL1 in establishment of the midbrain dopamine pathways, functions that are reinforced by evidence in other neuronal networks, yet roles that are also unique to this discrete population of neurons. Supported by spatial and temporal expression within the VM, and validated in CHL1 deficient mice, we: (i) identify a role for CHL1 in radial, and subsequent tangential migration of DA neuroblasts, (ii) demonstrate the influence of substrate-bound CHL1 on DA differentiation, and (iii) highlight the impact on CHL1 on the elongation and directional growth of DA axons towards their appropriate forebrain targets. In addition to expanding our knowledge of VM development, further studies, advancing on these findings, may unravel novel roles of CHL1 within neurological conditions in particular developmental disorders affecting midbrain DA neurons, as well as improve therapies for dopamine-related disorders, such as the ability of CHL1 to improve the yield, and subsequently plasticity, of DA neurons from stem cell populations for transplantation into the Parkinsonian brain.

## Electronic supplementary material

Supplementary Figure 1-3
