# Supplementary material for: Homophilic binding of the neural cell adhesion molecule CHL1 regulates development of ventral midbrain dopaminergic pathways
Source: Sci Rep. 2017 Aug 24;7:9368. doi: 10.1038/s41598-017-09599-y (PMC5570898; doi:10.1038/s41598-017-09599-y)
Supplement: Supplementary file 1 — Supplementary Figure 1-3 [file 41598_2017_9599_MOESM1_ESM.pdf]

Homophilic binding of the neural cell adhesion molecule CHL1 regulates development of ventral midbrain dopaminergic pathways

W.F. Alsanie[1], V. Penna[1], M. Schachner[2], L.H. Thompson[1], C.L. Parish[1]

[1] The Florey Institute of Neuroscience and Mental Health, The University of Melbourne, Melbourne, Australia. [2] Keck Center for Collaborative Neuroscience and Department of Cell Biology and Neuroscience, Rutgers University, NJ, USA.

Correspondence to: A/Prof Clare Parish
clare.parish@florey.edu.au
+613 9035 6526

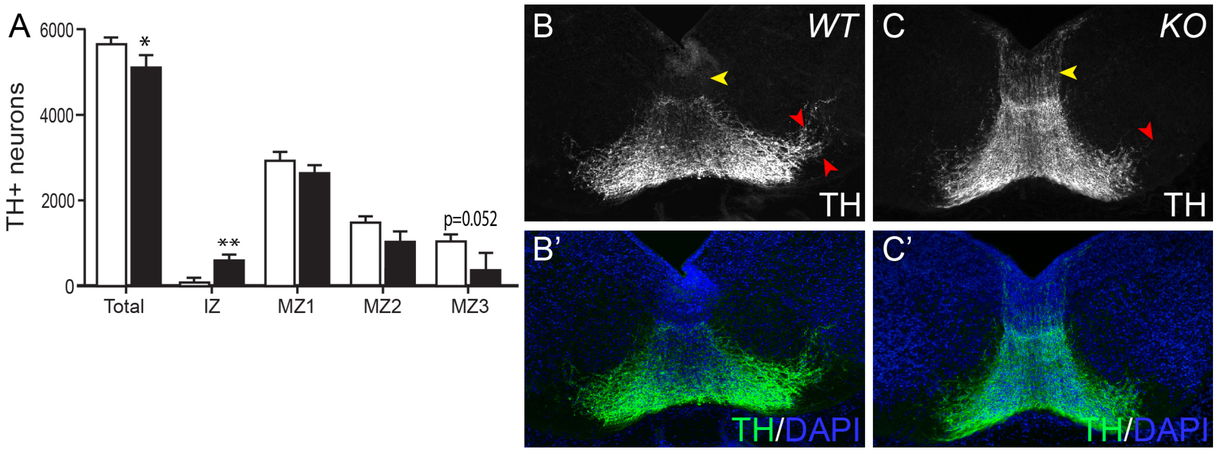

Supplementary Figure 1. CHL1 deficient mice display persistent defects in the positioning of TH+ progenitors/neurons and reduced differentiation. (A) Quantification of TH+ neurons in the VM of E14.5 WT (open bars) and CHL1 KO littermates (closed bars), and their distribution within the intermediate (IZ) and marginal zones (MZ1, MZ2, MZ3). (B) By the end of DA neurogenesis, at E14.5, CHL1 deficient mice show persistent defects in the radial and tangential migration of TH+ DA progenitors, as revealed by the increase in cells within the IZ (yellow arrow) and reduced cells within the lateral region of the MZ (red arrow), compared to (C) WT littermates. Data represents Mean $\pm$ SEM, * $p<0.05$, ** $p<0.01$.

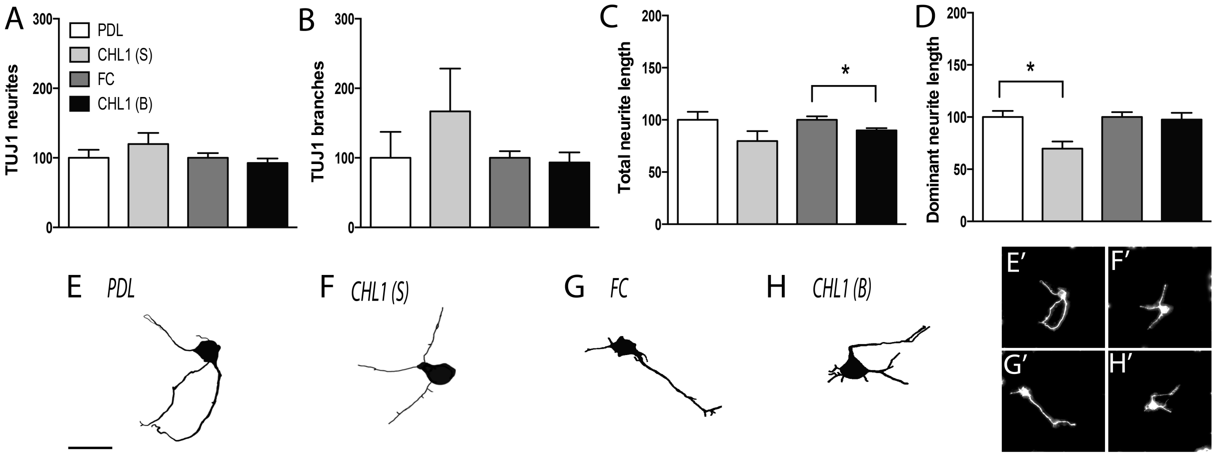

Supplementary Figure 2. CHL1 opposes neurites outgrowth of non-dopaminergic neurons within vmDA primary cultures. CHL1-(S) and CHL1-(B) do not have an effect on the number of neurites and branches in TUJ1-positive/TH-negative vmDA neurons isolated from E12.5 embryos (A, B). CHL1-(B) decreases the total neurites outgrowth in TUJ1-positive/TH-negative vmDA neurons isolated from E12.5 embryos (C). CHL1-(S) only decreases the length of the dominant neurites length in TUJ1-positive/TH-negative vmDA neurons isolated from E12.5 embryos (D). The immunocytochemistry images show the effect of CHL1 on TUJ1-positive/TH-negative vmDA neurons isolated from E12.5 embryos (E-H and E'-H'). N=5. Data represented as mean ± SEM. Scale bar = 20 um. * p=0.05, ** p=0.01, ***p=0.001, ****p=0.0001.

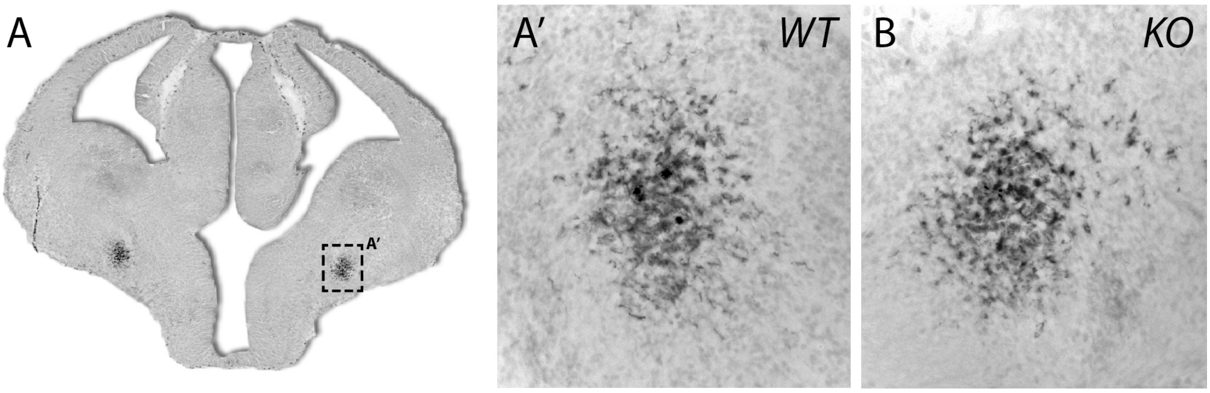

Supplementary Figure 2. CHL1 deficient mice show no gross defects in development of the midbrain dopaminergic pathways. (A) Coronal section of the E14.5 brain illustrating the rostro-caudal level at which gross assessments of TH+ fibre density and axonal fasciculation within the medial forebrain bundle were made. (A') Representative high power (40X) image of TH+ axons within the MFB of a WT and, (B) CHL1 KO embryo. Note no obvious differences in the density or distribution of TH+ fibers between the two genotypes.
